# Improving antibiotic prescribing for community-acquired pneumonia in a provincial hospital in Northern Vietnam

**DOI:** 10.1093/jacamr/dlab040

**Published:** 2021-05-16

**Authors:** Nga T T Do, Ryan Li, Huong T T Dinh, Huong T L Nguyen, Minh Q Dao, Trang N M Nghiem, Behzad Nadjm, Khue N Luong, Thai H Cao, Dung T K Le, Francoise Cluzeau, Chau Q Ngo, Hanh T Chu, Dat Q Vu, H Rogier van Doorn, C Michael Roberts

**Affiliations:** Oxford University Clinical Research Unit, Hanoi, Vietnam; Global Health and Development Group, Imperial College London, London, UK; Thanh Nhan Hospital, Hanoi, Vietnam; Thanh Nhan Hospital, Hanoi, Vietnam; Thanh Nhan Hospital, Hanoi, Vietnam; Oxford University Clinical Research Unit, Hanoi, Vietnam; Oxford University Clinical Research Unit, Hanoi, Vietnam; Nuffield Department of Clinical Medicine, University of Oxford, Oxford, UK; University College London Hospitals NHS Foundation Trust, London, UK; Clinical Services Department, MRC Unit The Gambia at The London School of Hygiene & Tropical Medicine, Banjul, The Gambia; Medical Services Administration, Ministry of Health, Hanoi, Vietnam; Medical Services Administration, Ministry of Health, Hanoi, Vietnam; Medical Services Administration, Ministry of Health, Hanoi, Vietnam; Global Health and Development Group, Imperial College London, London, UK; Bach Mai Hospital, Hanoi, Vietnam; Bach Mai Hospital, Hanoi, Vietnam; Hanoi Medical University, Hanoi, Vietnam; National Hospital of Tropical Diseases, Hanoi, Vietnam; Oxford University Clinical Research Unit, Hanoi, Vietnam; Nuffield Department of Clinical Medicine, University of Oxford, Oxford, UK; Essex Partnership University NHS Trust, Wickford, UK; UCL Partners, London, UK; Royal College of Physicians, London, UK

## Abstract

**Objectives:**

To test the effectiveness of a quality improvement programme to promote adherence to national quality standards (QS) for patients hospitalized with community-acquired pneumonia (CAP), exploring the factors that hindered improvements in clinical practice.

**Methods:**

An improvement bundle aligned to the QS was deployed using plan-do-study-act methodology in a 600 bed hospital in northern Vietnam from July 2018 to April 2019. Proposed care improvements included CURB65 score guided hospitalization, timely diagnosis and inpatient antibiotic treatment review to limit the spectrum and duration of IV antibiotic use. Interviews with medical staff were conducted to better understand the barriers for QS implementation.

**Results:**

The study found that improvements were made in CURB65 score documentation and radiology results available within 4 h (*P *<* *0.05). There were no significant changes in the other elements of the QS studied. We documented institutional barriers relating to the health reimbursement mechanism and staff cultural barriers relating to acceptance and belief as significant impediments to implementation of the standards.

**Conclusions:**

Interventions led to some process changes, but these were not utilized by clinicians to improve patient management. Institutional and behavioural barriers documented may inhibit wider national uptake of the QS. National system changes with longer term support and investment to address local behavioural barriers are likely to be crucial for future improvements in the management of CAP, and potentially other hospitalized conditions, in Vietnam.

## Introduction

Lower respiratory tract infections (LRTIs) are a leading cause of morbidity and mortality worldwide.^1^ In Vietnam, respiratory infections and TB were the fifth leading cause of death, responsible for 35 760 deaths (5.9% of deaths) in 2017.^2^ Among LRTIs, community-acquired pneumonia (CAP) is a common condition for hospital admission and antibiotic use.^3^ High levels of antimicrobial resistance coupled with a lack of agreed standards of care impact on both outcomes and antibiotic prescribing, promoting antimicrobial resistance (AMR).^4^ This, in conjunction with the availability of well-developed international guidelines based on well validated prognostic models,^5^^,^^6^ make CAP an ideal starting point to develop quality standards (QS) to improve care and control antibiotic prescribing, which is in line with the aim of the National Action Plan (NAP) for combating AMR in Vietnam. As in many low- and middle-income countries (LMICs), the Vietnamese health system faces both high levels of AMR and a lack of standardized care for treatment and infection control.^4^ These factors are likely to impact both clinical outcomes and antibiotic prescribing, with subsequent effects on AMR. Despite the existence of national guidelines on antibiotic use produced by the Vietnam Ministry of Health in 2015,^5^ they are not consistently used in the absence of a national implementation strategy.

In 2013, Vietnam became the first country in the Western Pacific Region to approve a NAP, which was developed in response to the call from the WHO to have a timely plan to deal with AMR. Since then, Vietnam has shown remarkable progress in creating mechanisms for collaboration across sectors to improve surveillance system and control antibiotic use under the One Health approach.^6^ In line with the aim of the NAP, and as a first phase of this project in 2016, QS for appropriate antibiotic use in CAP for adult patients in Vietnamese hospitals were developed jointly by the international arm of NICE in the UK and the Vietnamese QS Working Group with local experts representing clinical care and health policy.^7^ The QS were based on international clinical guidelines, adapted for Vietnam comprising five statements that set out the priority areas for improvement in CAP care (Table S1, available as Supplementary data at *JAC-AMR* Online).^8–13^ The process drew from previous experience of the Vietnam Ministry of Health (MoH) and NICE International to develop QS and an implementation plan for the hospital management of acute stroke in Vietnam.^14^ Before implementing the QS nationwide, they were piloted in a 600 bed provincial city hospital in northern Vietnam, to provide lessons that would inform wider implementation/scale-up. As a small change is more likely to succeed than trying to change the whole pathway at once, it was agreed with the hospital leadership to implement two of the five statements (QS1 and QS5). These two statements were felt to potentially impact on patient care and antibiotic prescribing through improving diagnosis in a timely fashion and limiting the duration of antibiotic therapy. The specific objectives of the pilot were to use a series of plan-do-study-act (PDSA) improvement cycles to change processes and management of patients and secondly to identify barriers and enablers of change.^15^

## Methods

### Study setting

The pilot was conducted in a provincial general hospital having 600 beds in the capital Hanoi. In particular, it was conducted in the three departments where the most CAP patients were treated: the outpatient department (OPD), internal medicine department (IMD) and emergency department (ED).

### Improvement process design

The quality improvement process was developed jointly by international and local experts representing clinical care and health policy. The process was divided into four phases (P1 to P4) over 10 months (from July 2018 to April 2019) following the PDSA cycle methodology^15^ (Figure S1), including: plan—consisting of baseline data collection and mapping of existing care pathways (P1); do—training about use of the new QS and QS implementation (P2); study—mixed-method assessment (P3); act—re-training with adjustment based on findings from P2, including experience sharing by local clinical champions and providing tools to facilitate recording of the quality indicators in the patient records, re-implementation and re-assessment (P4).^16^ Piloting these QS, we aimed to reduce unnecessary admissions and use of broad spectrum IV antibiotics using CURB65 (Table S2) and to limit the duration of antibiotic courses by regular review. Quality improvement was measured using predefined quality indicators (Table S3).

To inform interventions, the pre-pilot care pathway was mapped to the QS by reviewing national guidelines and internal protocols (baseline assessment). The process map and written management policies were then compared with those from a sample of patients who presented with CAP at either ED or OPD. Case notes were audited against both the agreed hospital pathway and policies and against the new QS.

To better understand the barriers to applying the QS to routine practice and to identify opportunities for behavioural change, qualitative data were also collected through formal in-depth interviews (IDIs) with doctors managing CAP patients and through observation of practice. Content of the IDIs included the strengths and weaknesses of the QS and contextual factors affecting the application of QS in routine practice. In total, six IDIs with four IMD doctors and two ED doctors were conducted. Each interview lasted 45–60 min. The key findings were summarized with an emphasis on lessons learned and considerations for scaling up QS implementation to national level.

### Strategy

Through PDSA cycles, we tested the impact of training interventions to stimulate adoption of the QS among healthcare workers managing CAP patients. The first training (P2) took place on three afternoons in July 2018. Participants included representative doctors from the OPD, IMD, Occupational Disease Department and ED; microbiologists and clinical pharmacists. Training was developed and delivered by a team comprising international and Vietnamese experts in infection management and antibiotic stewardship who provided international and local evidence behind aspects of the QS (e.g. CURB65 as a prognostic scoring system, rationale for the length of antibiotic courses and the potential for harm if courses are excessive). The trainees were then divided into groups to discuss the QS implementation plan and proposed solutions, along with their benefits to the hospital and patients. Recommendations proffered during the training included the need to: (i) facilitate documentation through template pages for the patient record that included a section for CURB65 scoring, history of antibiotic use and antibiotic therapy review; (ii) widely disseminate the QS to other doctors in the hospital who did not join the training, with support from hospital leadership and clinical champions; and (iii) enhance the role of the clinical pharmacist in controlling long-term antibiotic use (more than 7 days) through case notes audit. Based on findings from the post-training pilot, the second training was focused on deepening the trust of local doctors in the QS through sharing local evidence about CURB65 and discussing the bioavailability of oral versus IV antibiotics. The training was delivered by a local infectious disease specialist. Tools for facilitating documentation were also adjusted by replacing the template pages for patient records, which were found difficult to use, with ink stamps to indicate that CURB65 score and antibiotic review (including history of previous use and during inpatient stay) had taken place.

### Ethical considerations

Permission was obtained from relevant international Ethics Committee (OxTREC, Reference: 536-18) and the local ethics committee of the hospital where the pilot study was conducted (1108/BVTN-CDT). Written consent was obtained from participants in IDIs.

### Data analysis

Collected data were cleaned and entered into an electronic database (clinical research data management system, CliRES) and checked for quality by an independent data analyst. Descriptive data were summarized in median and IQR for skewed distributed data and proportion. Potential differences between phases were compared by χ^2^ test for categorical variables. A significant difference (*P *<* *0.05) was interpreted as a significant effect of the intervention bundle.

All interviews were recorded and transcripts were made and translated into English. Data from transcripts were analysed using qualitative content analysis. The codes were sorted into coding categories related to main themes, according to the procedures of content analysis. The analysis was facilitated by QRS International NVivo (v.12) software.

## Results

### Pre-interventions

**Figure 1.**
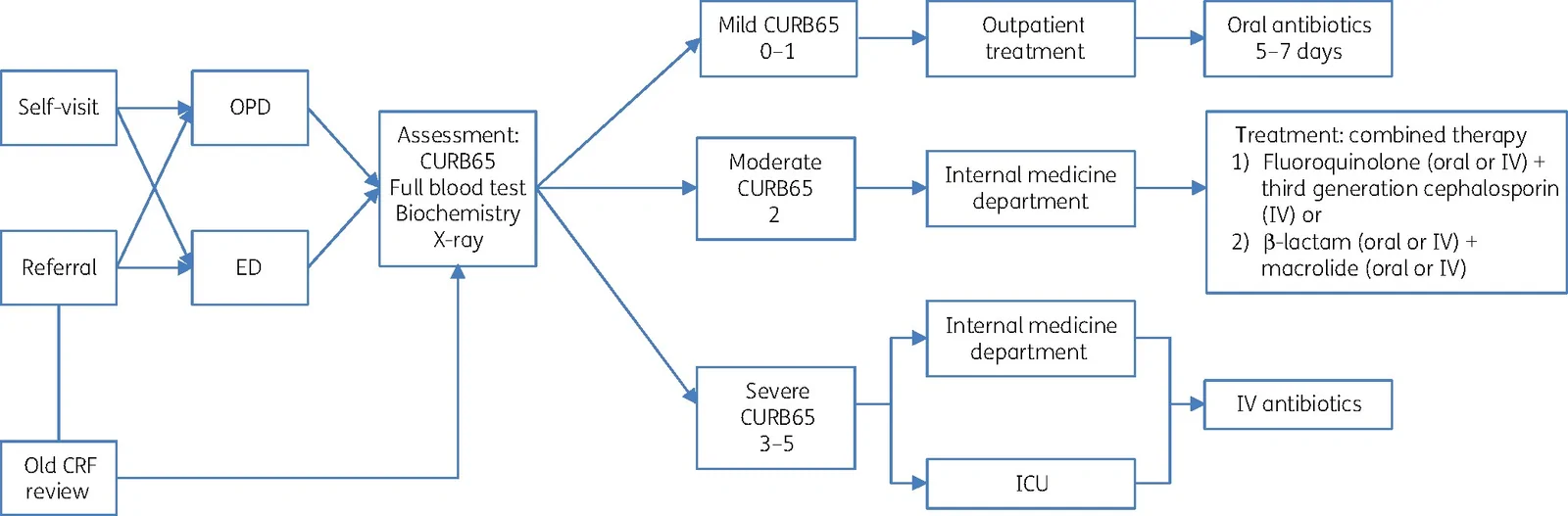
Mapping of pre-implementation care pathway for CAP patients. OPD, outpatient department; CRF, case record form; ED, emergency department.

The process mapping revealed that the patient journey (Figure 1) started either through self-presentation or referral from other healthcare facilities (district polyclinics or other hospitals) to either medical outpatients or the emergency department (ED) depending on acuity. Patients were then clinically assessed, including laboratory and radiography investigations as indicated. The baseline hospital pathway and policies already had much in common with the new QS. One key process measure, common to both, was that patient severity should be assessed using CURB65 scoring to guide admission decision-making.

Baseline case notes (30 in total) of CAP-confirmed patients admitted to the IMD in the preceding month were reviewed using International Classification of Diseases (ICD)-10 coding for pneumonia (J18); 2 cases were excluded due to incorrect coding (bronchitis diagnosis, not CAP) and the rest were reviewed against the QS (Table 1). The majority of the patients were male, the median age was 61 years, and most patients had at least one comorbidity, with hypertension and diabetes being the most prevalent at 38% and 19%, respectively. Whilst some practices adhered to hospital guidelines and the QS such as performing a chest radiograph, some other QS were neglected, particularly those relating to documentation of the CURB65 score to guide management and appropriate use of antibiotic therapy. There was a strong tendency to admit patients with low CURB scores and to treat with IV, rather than oral, antibiotics with long course duration periods (Table 1). These data were then presented to a focus group of medical staff from the hospital and a facilitated discussion took place in order to co-design a number of PDSA interventions that might improve compliance with the agreed quality standards. These interventions were then subjected to a pilot implementation programme.

**Table 1.** Assessment of the impact of change

Characteristics	Baseline (P1, *N *=* *28), *n* (%)	First pilot implementation (P2, *N *=* *70), *n* (%)	Second pilot implementation (P4, *N *=* *72), *n* (%)	Impact of change assessment		
				P2 versus P1, *P* value	P4 versus P1, *P* value	P4 versus P2, *P* value
Participant demographics						
age, years, median (IQR)	61 (44–81)	73 (47–85)	68 (53–80)			
male	19 (67.8)	38 (54.3)	39 (54.2)			
with comorbidity	21 (75.0)	46 (65.7)	56 (77.8)	**0.02**	0.97	0.16
History of antibiotic use						
yes	1 (3.6)	12 (17.1)	9 (12.5)	0.11	0.33	0.24
no	0 (0)	5 (7.1)	16 (22.2)	0.34	**0.01**	**0.04**
unknown	26 (92.8)	53 (75.7)	47 (63.3)	**<0.001**	**0.01**	0.38
Chest X-ray ≤4 h	11 (39.3)	62 (88.6)	55 (76.4)	**<0.001**	**0.001**	0.09
CAP confirmation ≤4 h	24 (85.7)	56 (80.0)	53 (73.6)	0.83	0.30	0.48
CURB65 score						
0–1	16 (57.1)	37 (52.8)	52 (72.2)	0.97	0.22	**0.002**
2	3 (10.7)	17 (24.3)	13 (18.0)	0.17	0.55	0.36
3–5	0 (0)	2 (2.8)	4 (5.6)	0.91	0.48	0.70
not documented	9 (32.1)	14 (20.0)	3 (4.2)	0.69	**<0.001**	**0.008**
Antibiotic treatment	27 (96.4)	67 (95.7)	71 (98.6)	0.99	0.99	0.97
initial IV	25 (92.6)	65 (97.0)	69 (97.2)	0.69	0.65	0.99
daily review to switch therapy	27/27 (100)	67/67 (100)	71/71 (100)	N/A	N/A	N/A
senior review (antibiotic use >7 days)	18/23 (78.3)	25/33 (75.7)	39/49 (77.5)	0.99	0.99	0.99

Significant differences are indicated in bold.

N/A, not applicable.

### Post-training implementation

Over the 6 weeks of the first pilot implementation post-QS training (P2), 70 CAP patients admitted to IMD (39 cases from ED, 31 cases from OPD) were reviewed. The results are presented in Table 1. The median age was 73 years, and the majority of patients were male. Most patients had at least one comorbidity with the most common conditions being hypertension (53%), followed by diabetes (23%). Documentation of pre-hospital antibiotic therapy and time to receive a chest radiograph both improved significantly but none of the other process measures mapped to the quality standards did.

### Pilot after training with adjustment

In the next 6 week round of implementation (P4), 72 CAP patients admitted to the IMD were reviewed against the QS (Table 1). The improvement in documentation of pre-hospital antibiotic therapy, reduced time to chest radiography and CAP confirmation within 4 h was maintained. In addition, documentation of the CURB65 score also improved significantly compared with baseline (*P *<* *0.001) and against the P2 performance (*P *=* *0.008). There was no significant change, however, in the admission appropriateness judged by the CURB65 score or in the use of antibiotic therapy. Non-severe CAP with low to moderate risk of mortality (CURB65 score ≤2) was predominant among hospitalized patients, while home-based care or short stay in hospital was recommended (Table 1). There were only a few cases [2.8% (2/70) cases in P2 and 5.6% (4/72) cases in P4] with higher mortality risk (CURB65 score 3–5) documented. No deaths were recorded during the pilot study.

### Potential barriers to implementation

The qualitative findings from the in depth interviews of clinical staff presenting ‘systemic’ and ‘behavioural’ barriers in QS implementation are summarized in Table 2. In terms of systemic barriers, interviewees shared that the payment mechanism of health insurance with limited payment for outpatient care, availability of antibiotics in the hospital and widespread self-medication among population were the factors associated with unnecessary hospitalization and IV antibiotic treatment. Representative quotes were expressed as below:

*‘Doctors just decide which group of antibiotics should be used for patients but the chosen route of administration depends on the availability of drugs in the pharmacy department.’*

(IMD doctor)

**Table 2.** Identified potential barriers in QS implementation

	Barrier
‘Systemic’ barriers	Limited payment for outpatient care, regulated by health insurance, leads to unnecessary hospitalization.
	Availability of antibiotics in the hospital.
	Widespread self-medication facilitated by unrestricted access to antibiotics prior to attendance, in the absence of local data on antibiotic resistance, leads to perception amongst doctors that IV antibiotics are needed.
‘Behavioural’ barriers	Senior doctors’ concerns about inadequate follow-up procedures and detection of complications for patients treated as outpatients.
	Senior doctors’ belief that CURB65 is of limited use in assessing the need for admission of elderly patients with multiple comorbidities.
	Doctors’ belief that IV antibiotics have better bioavailability and effectiveness (usually contrary to evidence).
	Senior doctors’ belief that studies on CURB65 from outside Vietnam are not relevant to the local population.
	Doctors’ perception that patients prefer hospital admission and IV treatment.

*‘70-80% of patients admitted to hospital had taken antibiotics at home and did not improve while their CURB65 score was not high. These cases were admitted to see why they did not recover.’*

(IMD doctor)

The second key area as a barrier to improvement was that of clinical behaviours. Doctors expressed their concerns about inadequate follow-up procedures and detection of complications for patients treated as outpatients. This concern was described as below:

*‘The patients might not be in severe enough condition for admission, but if we do not allow them to be admitted and they got worse, we would be in trouble.’*

(ED doctor)

Doctors also revealed their concerns about using CURB65 to assess severity to make a hospitalization decision due to lack of evidence for its effectiveness in the local context. In fact, CURB65 elements were documented, but its score was not applied in decision-making. Example quotes from interviewees are given below:

*‘I have not applied it as I think the CURB65 score is not very useful and there is not enough evidence for its reliability. So, we mostly base our decisions on chest X-ray or infection markers like WBC [white blood cell], CRP [C-reactive protein], PCT [procalcitonin] and other factors like age and underlying diseases.’*

(ED doctor)

*‘The CURB65 score is not a very good tool to be used in Viet Nam, especially not for young patients with severe lung injuries, alcohol addiction, drug users, HIV,… who often have a low CURB65 score. Some elderly patients also have a low CURB65 score but a very high PSI score. CURB65 does not take history of antibiotic use into consideration.’*

(IMD doctor)

Lastly, doctors’ perceptions about poorer bioavailability of oral versus IV antibiotic treatment and perceived patient’s expectation of IV therapy were among the clinical behavioural factors associated with over-prescription of injectable medicines.

*‘Depending on the patients’ physical condition and patients’ absorption capacity, but I mostly select IV antibiotics. It is not convenient to use oral antibiotics. I feel that it is more difficult to absorb oral antibiotics rather than IV antibiotics. The bioavailability of oral antibiotics is not as good as that of IV antibiotics.’*

(ED doctor)

*‘Hospitalized patients would like to be treated with intravenous antibiotics*. *We have to not only do our job but also satisfy patients, otherwise we do not follow the ethics.’*

(IMD doctor)

Promisingly, young doctors also expressed that they are willing to change their habit. They are aware of abusing antibiotics with unnecessary long courses, so they found the pilot QS useful to change current practice.

*‘In the past, we never thought of switching from IV to oral antibiotics, which I think was inappropriate. But now I have been trying to follow the QS and see that it is fine to switch from IV to oral antibiotics.’*

(IMD doctor)

## Discussion

This pilot demonstrated that, within an improvement programme designed to increase clinical practice compliance with national quality standards, clinical staff more closely adhered to appropriate documentation of information that should inform clinical decision-making as a result of the intervention but failed to use this data to change clinical behaviours to improve patient care. A recent systematic review of healthcare professional behaviour change interventions concludes that those that incorporate educational outreach, audit and feedback, and reminders are most likely to be successful.^17^ This current intervention included an education programme, an audit of current practice that was repeated at each of the two PDSA cycles and presented back to the clinical teams, the deployment of tools to prompt the recording and use of the data needed to support the QS, all of which map to this summary of best practice.

The reasons for the failure of the intervention to deliver improvements to patient care could be explained by the qualitative data collected from the in-depth interviews with clinical decision-making staff. Two key areas were described by staff as barriers to change, namely systemic factors and behavioural factors. The systemic factors are complex and inter-relate but are potentially soluble and associated with limited payment for outpatient care, regulated by health insurance. In 2017, approximately 80% of the total population of Vietnam was covered by health insurance.^18^ Most hospitals are reimbursed on a fee-for-service basis. Depending on the level of medical services and the insurance schemes (differentiated by types of employment), the health insurance scheme reimburses 80% to 100% of primary services’ cost and 40% to 100% of specialized services’ cost.^19^ These systemic barriers suggest that reforms to the health insurance payment mechanism, to better incentivize patient-centred care, are necessary to improve quality in line with the QS.^20^ Such reforms should lead to the development of viable outpatient and ambulatory care models that better meet population and health system needs. The second key area as a barrier to improvement was that of clinical behaviours. Although doctors were willing to improve the recording of information that could be used to make clinical decisions for patient care, personal prejudices prevented application. Younger doctors and trainees expressed a willingness to adopt national quality standards and to adhere to evidence-based instruments such as the CURB65 tool. In contrast, more senior doctors expressed a range of professional opinions based upon local knowledge and they felt international guidance and evidence are not relevant to the local population. These included fears that if patients managed as outpatients did not improve or felt worse they would lose confidence in the hospital and choose to reattend an alternative facility. Also noted were opinions to the effect that evidence on which to base the national QS was not relevant to their local situation and specifically that CURB65 did not apply in the context of multi-morbidity and to a Vietnamese population. To address this challenge, local data collection through clinical audit should be encouraged, to gain buy-in and acceptance of findings among healthcare workers by demonstrating the local applicability of these evidence-based standards of care. The feedback of such data to clinical teams is then best delivered through local senior clinical and policy champions,^21^^,^^22^ something that was not achieved in this study. Daily review case reports to adjust antibiotic therapy and documenting reasons associated with long-term antibiotic treatment (>7 days) have been implemented. In 2016, the Vietnam Ministry of Health issued a ‘Decision’ regarding implementing antimicrobial stewardship (AMS) at hospitals (MoH Decision No. 772).^23^^,^^24^ It is required that restricted antibiotics (e.g. carbapenems, colistin, fosfomycin) are reviewed and approved by an AMS team member when used. Doctors were aware of this process and, thus, it has likely impacted on the adoption of QS5 regarding daily review and senior review for long-term antibiotic treatment. The improvement programme led to some process changes, these, however, were not utilized by clinicians to change patient management in changing administration route from IV to oral antibiotics as well as reduce antibiotic duration. For healthcare workers, it is necessary to enhance training on updated evidence-based clinical guidelines and antibiotic stewardship to reduce inappropriate antibiotic use and limit the spread of antibiotic resistance in both inpatient and outpatient settings. Public education campaigns may also be useful if the perceptions that the public have strong preferences for hospital admission and IV rather than oral therapy are borne out by studies in this population.

Studies in other LMICs identified similar system-level and individual-level barriers in QS implementation, suggesting that such gaps and barriers are common in similar settings.^25^^,^^26^ Even the QS were designed by Vietnamese experts but were then rejected by the clinicians on the ground as not relevant to their population. Whilst specific solutions will differ by context, the dual approach of engendering local ownership of quality improvement activities whilst proactively understanding barriers to implementation of best practice to inform further improvement approaches will be applicable in all settings.

This is a limited study in one hospital with a relatively small population sample. Although the QS includes five statements, it was agreed with the hospital leadership to implement two of the five statements with the highest impact on antibiotic prescribing through improving diagnosis in a timely fashion and limiting the duration of antibiotic therapy. This selection aimed to minimize disruption to the health insurance reimbursement mechanism but might be associated with the limited change gained. Only doctors were interviewed to understand the barriers to change, and this may introduce a selection bias and affect the results as other healthcare workers (e.g. pharmacists and nurses) may have a different point of view. We believe, however, that doctors are the key decision-makers in the clinical pathway so provide greatest insight into barriers to change. Nevertheless, this is the first study of its kind in Vietnam and does highlight some interesting challenges to improvement that align with those found in similar health systems.

### Conclusions

In an environment naive to quality improvement, an evaluation of barriers to change is a key element of an improvement programme. Systemic barriers especially are likely to be specific to that particular national or regional context. Only by understanding the systemic and cultural barriers can a programme be appropriately designed and supported to succeed. If national quality standards are to be adopted at scale, then government policy should also be appropriately aligned to support the interventions recommended.

## Supplementary Material

dlab040_Supplementary_Data
