# Supplementary material for: Improving antibiotic prescribing for community-acquired pneumonia in a provincial hospital in Northern Vietnam
Source: JAC Antimicrob Resist. 2021 May 16;3(2):dlab040. doi: 10.1093/jacamr/dlab040 (PMC8127081; doi:10.1093/jacamr/dlab040)
Supplement: dlab040_Supplementary_Data [file dlab040_supplementary_data.docx]

**Supplementary data**

**Table S1**. Context specific quality standards

| QS1* | The initial thorough assessments including medical history, antibiotic use history, a thorough clinical examination and chest X-ray to make a diagnosis and prognostic scoring of CAP including timelines of CAP diagnosis confirmed within 4 hours and CURB65 guided management |
| --- | --- |
| QS2 | Sputum culture result based antibiotic treatment is recommended but without delaying the timely antibiotic therapy |
| QS3 | Measuring infection biomarkers (CRP, PCT) for optimising antibiotic therapy |
| QS4 | Restrict initial intravenous antibiotics for low and moderate severity CAP patients unless contraindicated |
| QS5* | Review of antibiotic therapy including daily review to consider switching therapy (admission route, dose, or discontinuing) and senior review for long-term antibiotic treatment of more than 7 days |

*QS: Quality standard, *Proposed for pilot implementation; CRP: C-reactive-Protein; PCT: Procalcitonin*

**Table S2**. Predefined quality indicators

| QS | Indicator |
| --- | --- |
| QS1. Timely diagnosis and CURB65 based management | - Proportion of adults diagnosed with CAP who received thorough assessment including antibiotic use history, clinical examination, chest X-ray and severity assessment using CURB65, a validated prediction rule for assessing clinical severity of CAP to guide management - Proportion of adults diagnosed with CAP who received a diagnosis within 4 hours of hospital presentation - Proportion of adults admitted to hospital in accordance with CURB65 score recommendation |
| QS5. Antibiotic treatment review | - Proportion of adult inpatients prescribed initial intravenous (IV) antibiotics in accordance with CURB65 score recommendation - Proportion of adult inpatients with CAP prescribed antibiotics who have a documented review for each day of antibiotic treatment to consider switching from intravenous to oral route, adjusting the dose, or discontinuing treatment and - Proportion of adult inpatients with CAP receiving antibiotics for more than 7 days who have a documented review by a senior clinician after 7 days of antibiotic treatment and have a clear rationale for continuing antibiotic treatment documented. |

**Table S3**. CURB65 Score and use as a triage tool

| CURB65 Score | Mortality Risk | Management implication |
| --- | --- | --- |
| 0-1 | 0.6-2.7% | Patient can safely be managed at home |
| 2 | 6.8% | Consider short observation stay in hospital early discharge |
| 3 | 14% | Admit to hospital |
| 4-5 | 27.8% | Admit to hospital and consider ICU escalation |
| 1 point for each criterion of:  Confusion; Blood Urea > 7mmol; Respiratory rate ≥ 30/min; Systolic blood pressure < 90 mmHg systolic *or* diastolic blood pressure ≤ 60 mmHg; age ≥ 65 years-old | | |


**Figure S1. Plan – Do – Study – Act (PDSA) cycle**

*Note: The PDSA cycle was adapted from National Health Service (NHS) guideline to test if piloted Quality Standards will lead to improvement in improving antibiotic prescribing for community acquired pneumonia (CAP) to inform large-scale implementation*.
